# High-Grade Atypical Teratoid/Rhabdoid Tumor in the Pituitary Region

**DOI:** 10.7759/cureus.54097

**Published:** 2024-02-12

**Authors:** Vincent S Alexander, Michael Ernst, Wang L Cheung, Alyssa N Obermiller, Catherine Mercado, Hermes Garcia, Kavita M Pattani

**Affiliations:** 1 Department of Research, Alabama College of Osteopathic Medicine, Dothan, USA; 2 Department of Head and Neck Surgery, Orlando Health Cancer Institute, Orlando, USA; 3 Department of Pathology, Orlando Health Cancer Institute, Orlando, USA; 4 Department of Radiation Oncology, Orlando Health Cancer Institute, Orlando, USA; 5 Department of Neurological Surgery, Orlando Health Cancer Institute, Orlando, USA

**Keywords:** inr-1, smarcb1, pituitary disorder, small blue cell tumor, atypical teratoid rhabdoid tumors

## Abstract

Atypical teratoid/rhabdoid tumors (AT/RTs) are embryological tumors of the central nervous system (CNS). They are typically found in children, with rare presentations in adults. We describe the presentation of an AT/RT in the pituitary region of a 37-year-old female. The patient presented with a two-week history of intractable cephalgia with sudden onset of monocular diplopia and left-sided cranial nerve VI palsy. The patient underwent transsphenoidal resection of their mass, which revealed the diagnosis. She then underwent systemic therapy with chemotherapy as well as radiation. She ultimately died 14 months after treatment completion due to unrelated events. The case highlights the rarity of AT/RT in adults, emphasizing the challenge of establishing standardized treatment protocols due to its rarity in adult presentations.

## Introduction

Atypical teratoid/rhabdoid tumors (AT/RTs) are malignant embryological tumors and one of the most common malignancies in the central nervous system (CNS) in children less than six months old [1]. In contrast, AT/RTs in adults are extremely rare. AT/RT of the pituitary region is even rarer and can present as an adenoma with common clinical symptoms of visual disturbances, pituitary dysfunction, and headaches [2]. We present a rare occurrence of an AT/RT in the pituitary region. There are less than 60 other documented cases of pituitary AT/RTs [2-3]. 

## Case presentation

**Figure 1:**
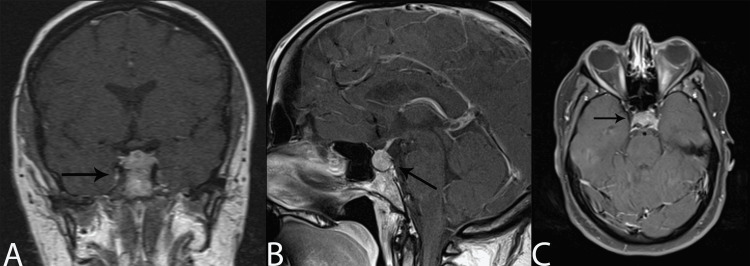
Brain MRI T1 post-contrast. (A) Coronal view: Sellar lesion with extension into the left cavernous sinus. (B) Sagittal view: Sellar lesion showing contrast enhancement extending into the left cavernous sinus in an infiltrative fashion. (C) Axial view: Sellar lesion with slight enlargement of the sellae. No suprasellar extension. MRI, magnetic resonance imaging

A 37-year-old female presented to the emergency department with a two-week history of intractable cephalgia with the onset of monocular diplopia. The patient also presented with left-sided cranial nerve VI palsy. The patient initially underwent a CT head that was reported as negative. However, a subsequent MRI was ordered for further evaluation. Upon completion of an MRI brain with contrast, she was found to have an enhancing pituitary mass measuring 17 mm x 12 mm x 12 mm, with extension into the left cavernous sinus, resulting in rightward infundibular deviation (Figure 1). The patient was initially suspected to have a pituitary adenoma. Given the onset of her symptoms, the patient was quickly scheduled for surgery. 

The patient was scheduled for an endoscopic transsphenoidal resection of the pituitary tumor. The surgery was completed under the coordination of Neurosurgery and Head and Neck (ENT) surgical teams. Intraoperatively, the tumor appeared inflammatory. Debulking was done, and adequate decompression was deemed appropriate. Intraoperative pathology consultation was inconclusive; thus, further resection was not pursued. However, the initial surgical pathology of the tumor revealed a high-grade neuroendocrine/primitive neuroepithelial tumor. Tumor studies and immunohistochemistry showed reticulin loss of normal pituitary architecture. Stains for adrenocorticotropin hormone, growth hormone, luteinizing hormone, thyroid stimulating hormone, follicle-stimulating hormone, and prolactin were all negative. CAM 5.2 was negative for fibrous bodies. Immunohistochemistry for Ki67 showed a proliferative index of 50%-60%. Tumor cells exhibited positivity for CD56, CD99, and synaptophysin, while testing negative for CD20, CD3, CD45, GFAP, MYP-D1, Myogenin, EMA, and S-100. These results align with the previously mentioned diagnosis. 

The patient was presented at a multidisciplinary cancer conference for consensus recommendations, with a recommendation for additional resection and postoperative radiation. Postoperative MRI of the brain showed postsurgical changes with residual enhancing pituitary/sellar lesion with left cavernous sinus involvement. 

**Figure 2:**
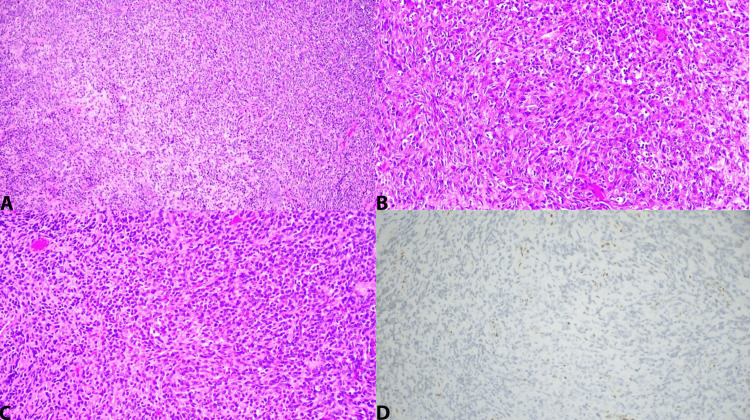
The tumor cells on hematoxylin and eosin (H&E) staining are present in sheets of small, round blue cells, consistent with a high-grade malignant neoplasm. In situ hybridization (ISH) for EBER is negative in tumor cells. Molecular studies are negative for the EWS gene. SMARCB1 mutation is present. As expected, INI1 immunohistochemical staining is lost in the tumor cells with a SMARCB1 mutation establishing the diagnosis of AT/RT. (A) H&E at x100; (B) H&E at x200 magnification; (C) H&E at x200 magnification; and (D) INI1 at x200 magnification. AT/RT, atypical teratoid/rhabdoid tumor; EBER, Epstein-Barr Virus Encoded RNA

**Figure 3:**
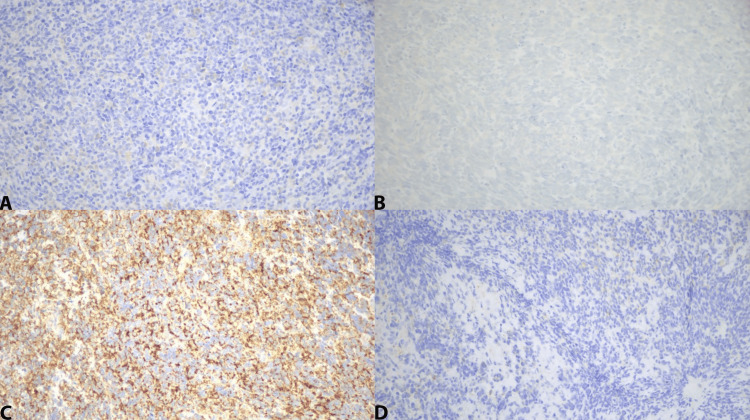
Immunohistochemical (IHC) staining is positive for CD99 and negative for p63, EMA, CAM5.2, MyoD1, myogenin, pancytokeratin AE1/3, and synaptophysin: (A) myogenin at x200 magnification; (B) P63 at x200 magnification; (C) CD99 at x200; (D) synaptophysin at x200.

**Figure 4:**
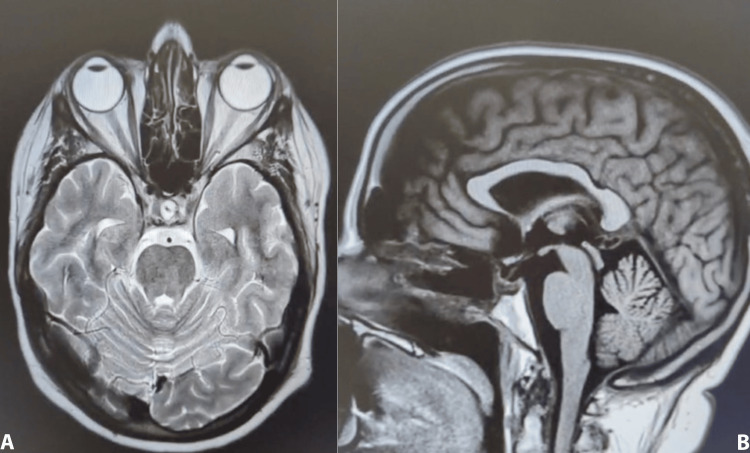
Brain MRI T2 post-contrast. Expected postoperative changes in the sellar region status post-transsphenoidal resection: (A) axial view; (B) sagittal view.

The patient returned to the operating room for further of a malignant sellar mass. Surgical pathology revealed a small blue cell tumor/primitive neuroectodermal Tumor (PNET) (Figure 2). Immunohistochemistry continued to show positivity with CD99, while testing negative for EMA, CAM 5.2, MYO D1, myogenin, and pancytokeratin AE1/3. Additionally, it remained positive for Synaptophysin (Figure 3). Immunostaining for p63 was negative. Fluorescence In Situ Hybridization (FISH) for Epstein-Barr Virus Encoded RNA (EBER) yielded negative results in tumor cells. Upon additional pathological review and immunohistochemistry, INI1 staining was lost in tumor cells, indicating a mutation in SMARCB1. Thus, the final diagnosis was made for an AT/RT. Postoperative CSF samples were obtained for flow cytometry with no monoclonal B-cell or blast population findings and no evidence of malignancy. Postoperative MRI imaging showed expected postsurgical changes and no evidence of gross residual tumor (Figure 4). 

She then underwent craniospinal proton beam radiation. She developed CSF rhinorrhea that required endoscopic transsphenoidal surgical repair with successful resolution. Treatment was completed with subsequent systemic therapy of ifosfamide, carboplatin, and etoposide. Unfortunately, she only completed three cycles due to the onset of severe myelosuppression and worsening symptoms, including headaches, nausea, and vomiting. The patient was without evidence of disease for 14 months before dying from circumstances unrelated to her disease course. 

## Discussion

AT/RTs are classified by the World Health Organization as a grade IV CNS tumor [4]. They are histologically similar to primitive neuroectodermal tumors but are distinctive due to the possible presence of rhabdoid cells and the described teratoid differentiation between various stages of mesenchymal and epithelial cells [1,4-5]. However, the diagnosis is confirmed with the SMARCB1 gene mutation (INR1 protein) and, in rare instances, the SMARC4 gene mutation [4]. The prognosis is suggested to be 20 months [6]. It should be noted that the vast majority of the documented AT/RTs of the pituitary/sellar region were of adult female patients [5][7]. The exact molecular mechanism has not been established. This is in contrast to pediatric patients, with the majority being male [1]. 

Gastberger et al. [8] discussed AT/RT subgroups: AT/RT-TYR, characterized by tyrosinase gene overexpression and associated with a younger patient demographic and better prognosis; AT/RT-SHH, marked by Sonic Hedgehog pathway gene activation, observed in slightly older patients with an intermediate prognosis; and AT/RT-MYC, identified by MYC gene amplification, linked to a more aggressive disease course and poorer prognosis [9]. This molecular stratification not only aids in understanding AT/RTs' diverse clinical presentations but also points toward potential targeted therapies. A potential role for immunotherapy as a treatment modality is being explored [10]. 

There has been some success in multimodality treatment protocols with initial surgical resection and subsequent radiotherapy and chemotherapy [2,7,11]. Further study with sufficient sample data is needed for proper treatment protocols and recommendations. 

## Conclusions

This clinical presentation of a 37-year-old female patient with an AT/RT in the pituitary region underscores the diagnostic and therapeutic intricacies associated with such an aberrant neoplasm. This case accentuates the need for further development in targeted therapies to ameliorate prognoses in AT/RT, particularly within adult demographics where its incidence is exceptionally uncommon, and the prognosis is poor. 

Given the rarity of AT/RTs, there is a clear paucity of standardized treatment guidelines and recommendations for adults, given the rarity of presentation. However, there is continued effort to explore treatment options and new avenues for stratification through genetic analysis. 
